# Targeting Bone Tumours with 45S5 Bioactive Glass

**DOI:** 10.3390/ijms251910830

**Published:** 2024-10-09

**Authors:** Joerg Fellenberg, Sarina Losch, Marcela Arango-Ospina, Nina Hildenbrand, Elena Tripel, Lingyun Deng, Tobias Renkawitz, Fabian Westhauser, Burkhard Lehner, Aldo R. Boccaccini

**Affiliations:** 1 Research Centre for Molecular and Regenerative Orthopaedics, Department of Orthopaedics, Heidelberg University Hospital, 69118 Heidelberg, Germany; 2 Institute of Biomaterials, Department of Materials Science and Engineering, University of Erlangen-Nuremberg, 91058 Erlangen, Germanyaldo.boccaccini@fau.de (A.R.B.)

**Keywords:** osteosarcoma, chondrosarcoma, giant cell tumour of bone, bioactive glass, therapy, chorioallantoic membrane assay

## Abstract

Despite advances in treatment modalities, bone tumour therapies still face significant challenges. Severe side effects of conventional approaches, such as chemo- and radiation therapy, result in poor survival rates and high tumour recurrence rates, which are the most common issues that need to be improved upon. The aim of this study was to evaluate the therapeutic properties of 45S5 bioactive glass (BG) for targeting bone tumours. The viability of the cells derived from osteosarcoma, chondrosarcoma, and giant cell tumours was significantly reduced in the presence of 45S5-BG. In contrast, the viability of non-malignant osteoblast-like cells, chondrocytes, and bone marrow-derived stromal cells was not or only slightly affected. While alterations to the particle surface induced by heat treatment, acid etching, or incubation in a simulated body fluid had only minor effects on cytotoxicity, reducing the particle size or sintering the material significantly improved the cytotoxic effect of 45S5-BG. Further, using a chicken chorioallantoic membrane assay, the co-transplantation of 45S5-BG resulted in a significant reduction in tumour formation in vivo. Given the known positive effects of BGs on bone regeneration, our findings suggest that 45S5-BG holds great potential for the development of new and effective bone tumour therapies, with minimal side effects on non-malignant cells and simultaneous contribution to bone healing.

## 1. Introduction

Bioactive glasses (BGs) are synthetic, amorphous, and biocompatible materials with a characteristic network structure that is composed of three distinct components. The network formers build up the basic structure and determine the type of BG with silica (SiO_2_), being the basis of silicate glasses, boron trioxide (B_2_O_3_) of borate glasses, and phosphorous pentoxide (P_2_O_5_) of phosphate glasses. The network modifiers, including CaO, Na_2_O, and others, change the BG structure by forming ionic bonds with the oxygen atoms of the network formers and largely determine the BG properties. The third component of BGs are the intermediate oxides, which are either network formers or modifiers. They are also called doping agents and allow for a further modulation of the network stability and the dissolution behaviour, thereby influencing the bioactivity of the BGs. Common intermediate oxides include Al_2_O_3_, MgO, Sb_3_O_2_, ZrO_2_, TiO_3_, PbO, and ZnO [1,2,3,4].

The outstanding feature of BGs that makes them versatile and successful materials for a wide range of medical applications such as tissue engineering, bone repair, and wound healing is the unique combination of bioactive properties that can be modulated by changing the BG composition. In contact with biological fluids, BGs form a hydroxyapatite-like surface layer that promotes a very strong bonding to soft and hard tissues including bone [5]. When BGs are used for bone repair, the subsequent attachment of osteoblast progenitor cells and their BG-induced differentiation facilitates the formation of new bone. Aside from these osteoconductive and osteoinductive properties, the beneficial effects of BGs have also been shown concerning angiogenesis, soft tissue repair, and neocartilage formation [6]. Another important feature of BGs is their ability to dissolve in aqueous solutions and to release ions, with the type and amounts of ions being determined by the BG composition. The combination of surface reactivity and the therapeutic ion release that has been shown to influence proliferation, mineralization, gene expression, and many other cellular functions [7] finally determines the bioactivity of the BG [8,9].

Although most clinical applications of BGs are found within the area of tissue engineering and bone repair, there is increasing evidence that BGs are also promising candidates for the development of new therapeutic strategies for the treatment of cancer [10,11]. One possible application of BGs in cancer therapy is the use of magnetic BGs for the induction of a hyperthermia effect. Magnetic glass ceramics are implanted near the tumour where they are immobilized through the apatite-mediated binding to the tissue. Exposition to an external, alternating magnetic field results in the development of heat and finally death of the tumour cells [12,13]. Bioactive glasses can further be used as carriers of tumour-targeting drugs including cytostatic drugs like doxorubicin and methotrexate as well as kinase inhibitors and even nucleic acids for gene therapy approaches. For this purpose, mesoporous BG nanoparticles are particularly suited, since they can easily be loaded with drugs and can even be internalized by the cells [14]. BGs can be further doped with bioactive ions like iron, lithium, or zinc to achieve specific therapeutic effects. Gallium-doped BGs, for example, have been shown to specifically target human osteosarcoma cells without local or systemic toxicity, while osteoblasts were not negatively influenced [15].

In most of the above-mentioned applications, BGs are mainly used as a shuttle for the transport of therapeutically active substances to the tumour and to anchor them to the tissue. Less is known about the direct effects of the BGs itself. Nano- and micro-sized BG particles and monodisperse silica nanoparticles have been shown to mediate the cytotoxic effects in endothelial cells and osteoblasts in a size-dependent manner [16,17]. In osteoblasts, the cytotoxic effect was initialized by an internalization of the BG particles into the cells, followed by a size-dependent localisation that determined the degree of cytotoxicity [16].

In our previous studies, we demonstrated a potent cytotoxic effect of the 45S5-BG towards neoplastic stromal cells that had been isolated from giant cell tumours of the bone (GCTB), while the viability of non-neoplastic mesenchymal stromal cells was not negatively affected [18,19]. 45S5-BG is considered the ancestor of silicate-based-bioactive glasses and was synthesized by Hench et al. over 50 years ago [20]. Since its FDA approval in 1993, 45S5-BG has been used in numerous clinical applications, primarily for the treatment of bone defects. Semi-malignant GCTBs are a rare and heterogeneous group of bone neoplasms that include tumours as diverse as the highly malignant osteosarcoma (OS) and the cartilage-forming chondrosarcoma (CS). For the semi-malignant GCTB, intralesional resection of the tumour tissue is the standard of care but is, however, associated with a very high recurrence rate ranging from 20 to 65% [21,22]. While surgical resection is often the only treatment option for GCTB and for the generally chemoresistant CS, current OS therapy is mainly based on chemotherapy. However, the five-year survival rate for CS and OS patients, especially those with metastatic or recurrent disease is still very low (20–40%), and improvements in bone tumour therapies and prognosis over the last decades have been very limited [23,24]. New therapeutic strategies that replace or complement conventional therapies are urgently needed. Ideally, such therapies should also allow the simultaneous treatment of bone defects caused by bone tumour growth and surgery.

Our previous data on GCTB cell lines, showing a tumour cell-specific cytotoxicity of BGs, together with the known beneficial effects of BGs on tissue regeneration and bone repair, suggest that the use of BGs may represent such an approach to reduce tumour recurrence while reconstructing bone defects. In the present study, we demonstrate, for the first time, that the cytotoxicity of BGs is not limited to GCTBs but can be observed independently of the tumour type in multiple bone tumour cell lines. This finding suggests the potential for a therapeutic use of BGs in the treatment of bone tumours in general. Furthermore, the comparison with non-malignant primary cells indicated that the therapeutic treatment would result in no or only minimal adverse effects. Additionally, the findings of our study suggest that the cytotoxic effect of the BG particles can be further enhanced by surface manipulation.

## 2. Results

**Figure 1:**
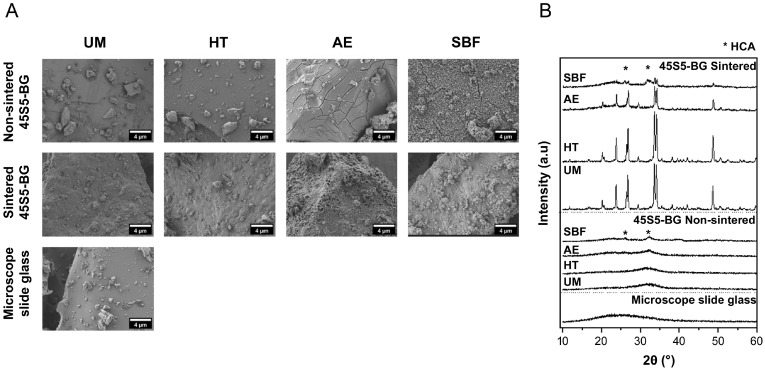
Characterization of glass particles. (**A**) Qualitative evaluation of the surface of unmodified (UM) glass particles and particles after heat treatment (HT), acid etching (AE), and incubation in simulated body fluid (SBF) by scanning electron microscopy (SEM). Images of particles in the range of 25–45 µm were considered for all groups. (**B**) XRD patterns of sintered 45S5-BG particles, non-sintered 45S5-BG particles, and microscope slide glass, before and after treatment. (HCA = hydroxycarbonate apatite).

Bioactive glass particles were separated into different size ranges and evaluated in terms of their surface morphology, particle size distribution, and crystallinity. Figure 1A shows SEM images of the glass particles in which the surface can be observed. As a result of the milling process, smaller fragments of glass were observed on top of all the particles and were not removed by the vibration during sieving. Notably, a smooth surface was observed between the non-sintered unmodified 45S5-BG and microscope slide glass, whereas for the sintered unmodified particles, a rougher surface was visible. After the heat treatment, there was no significant difference within the sintered group, and a slightly rougher surface was observed for the non-sintered BG particles compared to the unmodified particles. However, a clear difference between the sintered and non-sintered groups was observed after the acid etching and immersion of the particles in the SBF solution. In the non-sintered group, cracks were present throughout the whole volume of the BG particles, while porous regions were observed on the surface of the sintered particles. These differences may be attributed to the presence of crystalline phases formed after the sintering process, leading to different etching patterns on the surface. Figure 1B depicts the X-ray diffractograms of the glass particles, in which crystalline peaks corresponding to combeite-type phases (Na_2_CaSi_2_O_6_ and Na_2_Ca_2_Si_3_O_9_) were present in the sintered particles [25]. After the acid etching process, the intensity of about all crystalline peaks significantly decreased, which might be due to the dissolution process of the glass. In contrast, BG particles immersed in SBF exhibited the formation of apatite-like structures on the surface, which is characteristic of 45S5-BG and may correspond to the deposition of hydroxycarbonate apatite (HCA), which was detected in the XRD patterns as broad peaks around 26° and 32° [26]. This effect was observed for both sintered and non-sintered particles. In particular, the SEM images show that the HCA layer seemed more homogeneous in the non-sintered group than in the sintered particles, which was also related to the partially crystallized nature of the sintered group. It has been reported that crystallization in bioactive glasses could lead to a reduction or delay in the bioactive behaviour or apatite-layer formation; however, it has been demonstrated that, for the 45S5-BG composition, this process is not hindered by the presence of crystalline phases [27,28].

The characteristic parameters of particle size distribution analysis are shown in Table 1 for each analysed particle range, with the particle size distribution referring to a specific percentile of the particle size distribution curve. This means that 10% of the particles are smaller than the D10 value, 50% are smaller than the D50 value and 90% are smaller than the D90 value. No significant difference was found between the sintered and non-sintered particles, whereas lower values were measured for the particles derived from the microscope slide glass.

The primary objective of this study was to evaluate the cytotoxicity of 45S5-BG against bone tumour cells and to demonstrate its tumour cell-specific effects. To address the diversity of bone tumours and the known inter-patient variability within each type, this study includes 15 tumour cell lines from three different bone tumour entities. Primary cell lines were isolated from the GCTB and CS patients who underwent surgery at the Orthopaedic University Hospital in Heidelberg, whereas established cell lines were used for investigating the OS cells. To demonstrate tumour cell-specific effects, it is crucial to compare the results with appropriate non-neoplastic control cells. Therefore, we included 15 additional cell lines in the study, which consisted of osteoblast-like cells (HOB), chondrocytes (CHO), and bone marrow-derived mesenchymal stromal cells (BMSC) (*n* = 5 each). They represent the most abundant cell types in healthy bone and would therefore also be affected by a possible therapeutic approach using BGs.

**Figure 2:**
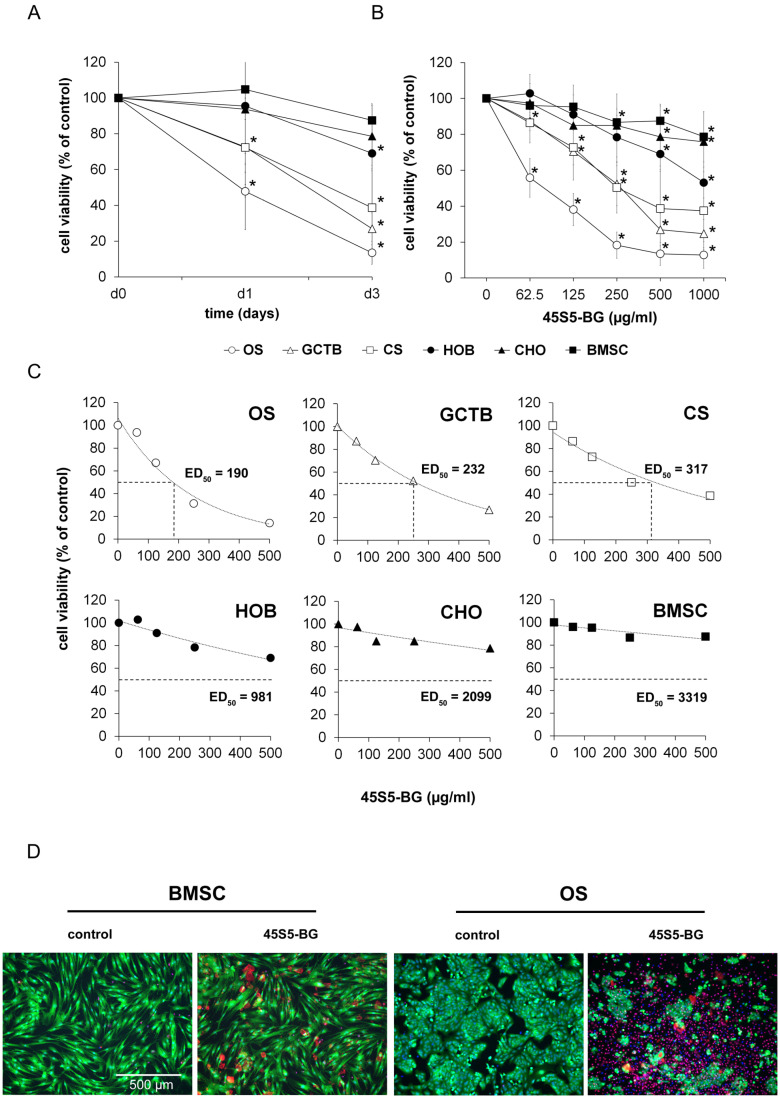
Cytotoxic effects of 45S5-BG. Tumour cell lines (OS, GCTB, and CS) (*n* = 5 each) and control cell lines (HOB, CHO, and BMSC) (*n* = 5 each) were (**A**) treated with 45S5-BG at a concentration of 500 µg/mL for 1 and 3 days or (**B**) treated for 3 days with 45S5-BG at different concentrations ranging from 62.5 to 1000 µg/mL. Cell viabilities were analysed by WST-1 assay, and data are presented as mean percentage viability compared to untreated cells with standard deviation. All experiments were performed in duplicates (* *p* < 0.05). (**C**) For the calculation of the ED_50_ values, tumour and control cell lines were treated with increasing concentrations of 45S5-BG, ranging from 31.25 µg/mL to 500 µg/mL for 3 days, before cell viability was quantified by WST-1 assay. ED_50_ values were calculated or extrapolated from the obtained trend line of the measured data. (**D**) Fluorescent staining of OS and BMSC cells with and without treatment with 45S5-BG for 3 days. The nuclei of all cells were visualised using Hoechst 33342 (blue). Viable cells were stained with calcein-AM (green) and the nuclei of dead cells were stained with propidium iodide (red) but appear purple due to the additional Hoechst 33342 staining. The BG particles were also partially stained by propidium iodide (red).

In a first step, we treated these cell lines with 45S5-BG at a concentration of 500 µg/mL and analysed the cell viability after 1 and 3 days. In these experiments, sintered 45S5-BG particles with a size of 65 ± 20 µm were used. All tumour cell lines showed a rapid and time-dependent decrease in the cell viability that was already significant after 24 h of treatment, with the OS cells being the most sensitive. In contrast, the control cell lines’ response was much less sensitive to the 45S5-BG treatment (Figure 2A). The observed cytotoxic effect increased with the concentration of 45S5-BG applied to the cells. A concentration of 62.5 µg/mL was found to be sufficient to negatively impact tumour cell viability, while much higher doses were necessary to influence the control cells (Figure 2B). To directly compare the sensitivities of the different cell types to the 45S5-BG treatment, we calculated the 45S5-BG concentrations required to inhibit cell viability by 50% after 3 days of incubation (ED_50_ values). While 190 to 317 µg/mL 45S5-BG was sufficient to kill 50% of the tumour cells, the ED50 values for the control cells had to be extrapolated from the data. The calculated values ranged from 918 to 3319 µg/mL, highlighting the marked differences in the sensitivity of the tumour and control cells to 45S5-BG (Figure 2C). To confirm the cytotoxicity of 45S5-BG particles and to exclude the possibility of a cytostatic effects, we conducted a fluorescent live/dead staining. Viable cells were stained with calcein-AM (green) while dead cells were stained with the cell membrane-impermeable propidium iodide (red). In order to distinguish between the cell nuclei of dead cells and the BG particles that also bind propidium iodide, an additional staining procedure was performed using Hoechst 33342 (blue). As a consequence, the nuclei of dead cells appear purple and can be distinguished from the red BG particles. While the number of viable control cells remained largely unchanged, the number of viable tumour cells decreased significantly after 3 days. Accordingly, the number of dead tumour cells rapidly increased after BG treatment. Representative images of OS and BMSC cells are shown in Figure 2D.

**Figure 3:**
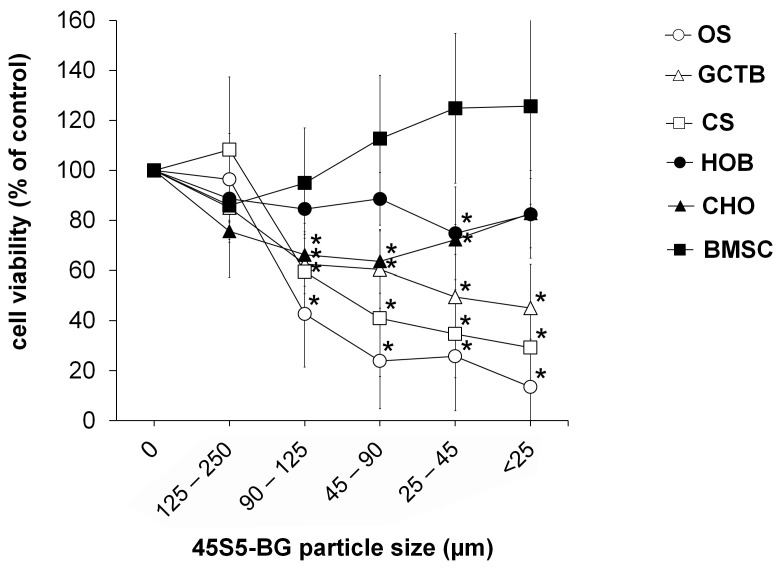
Impact of 45S5-BG particle size on cytotoxicity. Tumour cell lines (OS, GCTB, and CS) (*n* = 5 each) and control cell lines (HOB, CHO, and BMSC) (*n* = 5 each) were treated with 45S5-BG (500 µg/mL) at the indicated particle sizes. Cell viability was quantified by WST-1 assay after 3 days of incubation. Data are presented as mean percentage viability compared to untreated cells with standard deviation. All experiments were performed in duplicates (* *p* < 0.05).

In order to investigate the impact of the particle size on the cytotoxic effect of 45S5-BG, five different size fractions were produced by grinding and subsequent sieving of 45S5-BG. We observed a clear relationship between particle size and cytotoxicity, with the smallest particles having the greatest effect. These data also confirmed the observed predominant effect of 45S5-BG on the tumour cell viability compared to the analysed control cells (Figure 3).

**Figure 4:**
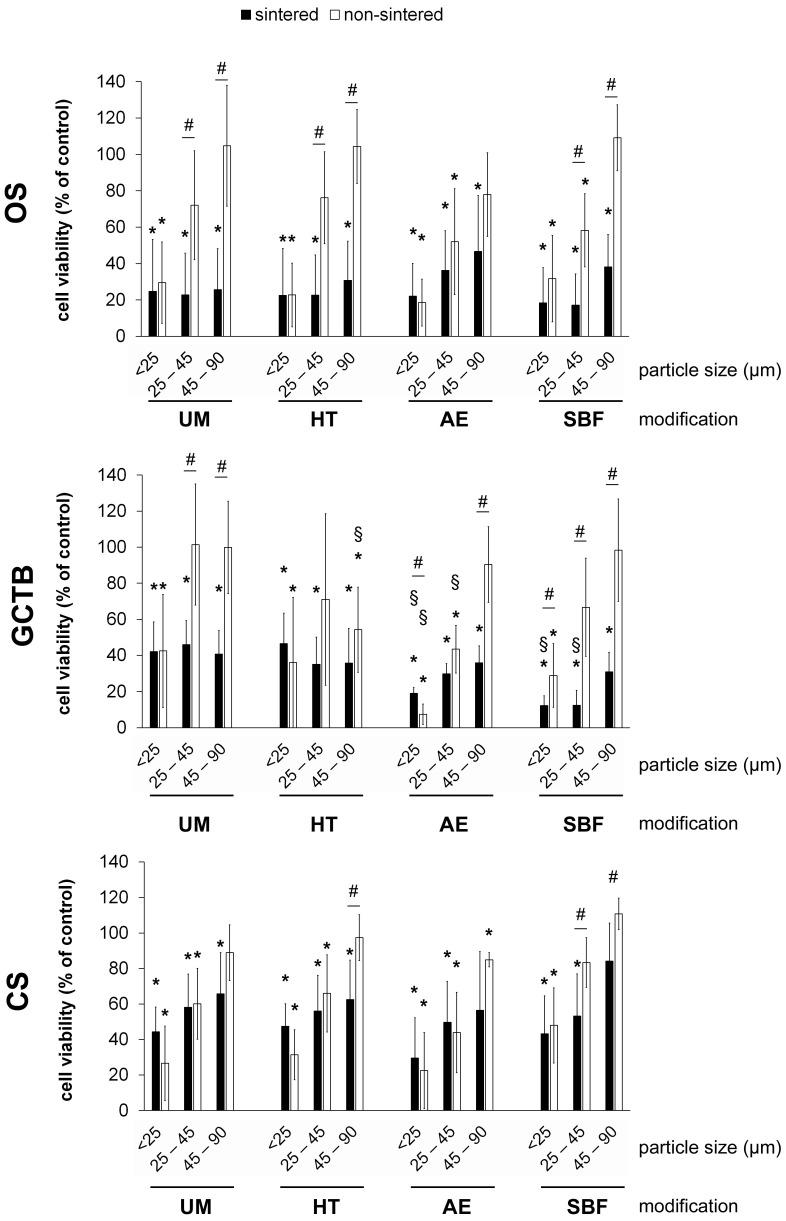
Influence of BG surface manipulation on cytotoxicity. OS, GCTB, and CS cell lines (*n* = 5 each) were treated with 45S5-BG variants that were either unmodified (UM), heat-treated (HT), treated by acid etching (AE), or incubated in simulated body fluid (SBF). All 45S5-BG variants were used in sintered and non-sintered form at a concentration of 500 µg/mL and in the indicated particle sizes. Data are presented as mean percentage viability compared to untreated cells with standard deviation. All experiments were performed in duplicates (* *p* < 0.05 compared to untreated cells; # *p* < 0.05 sintered compared to non-sintered BG; § *p* < 0.05 compared to unmodified 45S5-BG (UM)).

These data, together with our previous observations showing no improvement in 45S5-BG mediated cytotoxicity after modulation of the chemical composition [19], suggest that physical parameters may play a critical role in mediating the cytotoxic effects. Thus, we aimed to alter the surface properties of the 45S5-BG particles by different approaches including heat treatment, acid etching, and immersion in simulated body fluid (SBF). The cytotoxic effect of these 45S5-BG variants towards the various tumour cell lines was assessed using the WST-1 assay after three days of treatment. Based on the results described above, all BG variants were tested in the three most effective particle sizes < 25 µm, 25–45 µm, and 45–90 µm and at a concentration of 500 µg/mL. Of all the BG modifications that were investigated, sintering of the glass particles showed the strongest impact on cytotoxicity. All variants of sintered 45S5-BG significantly reduced cell viability at all the tested particle sizes. In contrast, non-sintered BGs reduced cell viability to a much lesser extent. Although sintered and non-sintered particles smaller than 25 µm were almost equally effective, differences became visible at larger particle sizes, with the non-sintered particles > 25 µm being less effective or not effective at all. These effects were observed for all tested 45S5-BG surface modifications and all tested tumour cell lines (Figure 4). Regarding the analysed BG surface modifications, heat treatment did not result in any changes compared to unmodified 45S5-BG. However, acid etching and incubation of the BG particles in SBF showed a trend towards increased cytotoxicity at smaller particle sizes that was significant in the case of GCTB cells (Figure 4).

**Figure 5:**
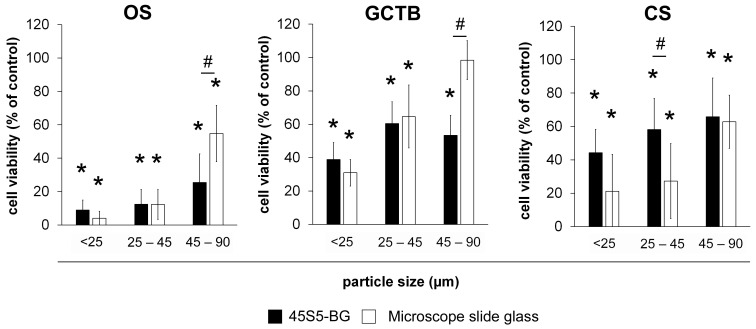
Comparison of cytotoxicity between 45S5-BG and microscope slide glass. OS, GCTB, and CS cell lines (*n* = 5 each) were treated with 45S5-BG or microscope slide glass particles at a concentration of 500 µg/mL for 3 days before cell viability was quantified by WST-1 assay. Data are presented as mean percentage viability compared to untreated cells with standard deviation. (* *p* < 0.05 compared to untreated cells; # *p* < 0.05 45S5-BG compared to MS glass).

To further investigate the importance of the bioactivity of the BG for the cytotoxic effect, we compared unmodified 45S5-BG with bio-inert microscopic slide (MS) glass particles of the same particle size. Interestingly, the viability of all investigated tumour cells was inhibited by MS glass to a comparable extent as 45S5-BG, except for particles larger than 45 µm, where 45S5-BG was generally more effective. From these data, we conclude that bioactivity is not a necessary requirement for cytotoxicity (Figure 5).

The impact of 45S5-BG on the development and growth of bone tumour cell lines in vivo was investigated using a chorioallantoic membrane (CAM) assay that we previously optimised for the use with bone tumour cells [29]. The CAM of fertilised chicken eggs is a highly vascularised membrane that mimics, together with extracellular matrix proteins, the physiological tumour environment. Furthermore, the natural immunodeficiency of the chick embryo, that lacks cell-mediated immunity until day 14, further facilitates tumour growth and development. In the first step, we characterised the tumour xenografts that we obtained after transplantation of the OS, GCTB, and CS cells onto the CAM. A Lens Culinaris Agglutinin staining was performed to specifically detect chicken cells, while human cells were targeted using in situ hybridisation with a DNA-probe specific for human ALU sequences.

**Figure 6:**
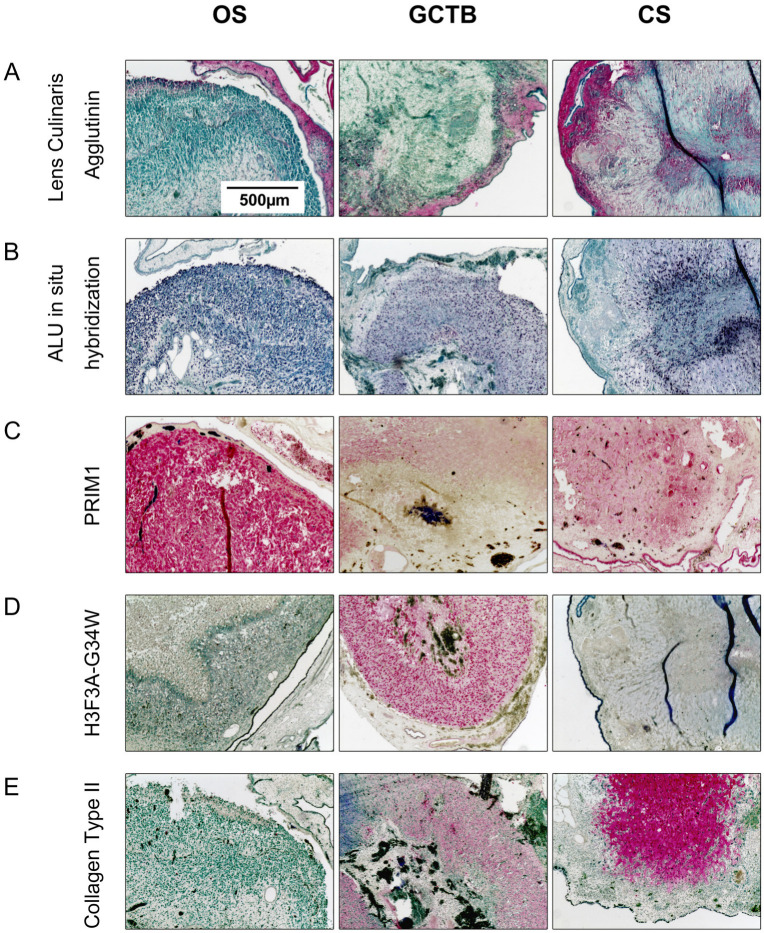
Immunohistochemical characterization of tumour xenografts. Sections from paraffin-embedded tumour xenografts were (**A**) stained with Lens Culinaris Agglutinin (red), (**B**) hybridised with a human ALU DNA probe (dark blue), or immunohistochemically analysed for the expression of (**C**) Prim1 (red), (**D**) H3F3A-G34W (red) or (**E**) Collagen type II (red). Sections were counterstained with Methyl Green. Representative images are shown for each tumour type.

While the red-coloured chicken cells were found almost exclusively in the CAM surrounding the tumour, the tumour mass consisted mainly of dark-blue-coloured human cells. Thus, both the Lens Culinaris Agglutinin staining and ALU in situ hybridisation confirmed the human origin of the cells within the tumour tissue that was surrounded by the chicken CAM. (Figure 6A,B). To detect the presence of specific tumour markers within the xenografts, we performed the immunohistochemical detection of PRIM1 (DNA primase polypeptide 1), which is known to be overexpressed in OS, H3F3A-G34W, which detects a mutation within the histone H3.3 that is characteristic for GCTB, and Collagen Type II, which is highly expressed in cartilage tissue and CS. OS tumour xenografts showed a strong PRIM1 expression, which was less evident in GCTB and CS. While only the GCTB-derived tumours displayed the characteristic expression of mutated H3F3A, the CS-derived tumours showed the expected strong expression of Collagen Type II. These data indicate that all the xenografts maintained the characteristics of the original tumours during the CAM assay (Figure 6C–E).

The CAM assay was then used to investigate the impact of 45S5-BG on tumour development in vivo. Tumour cells were suspended in cell culture medium with increasing concentrations of 45S5-BG and transplanted onto the CAM of fertilised chicken eggs (*n* = 20 in each experimental group). Sintered 45S5-BG particles were used for these experiments as a mixture of the particle sizes that were most effective in the in vitro experiments ranging from <25 µm to 90 µm.

**Figure 7:**
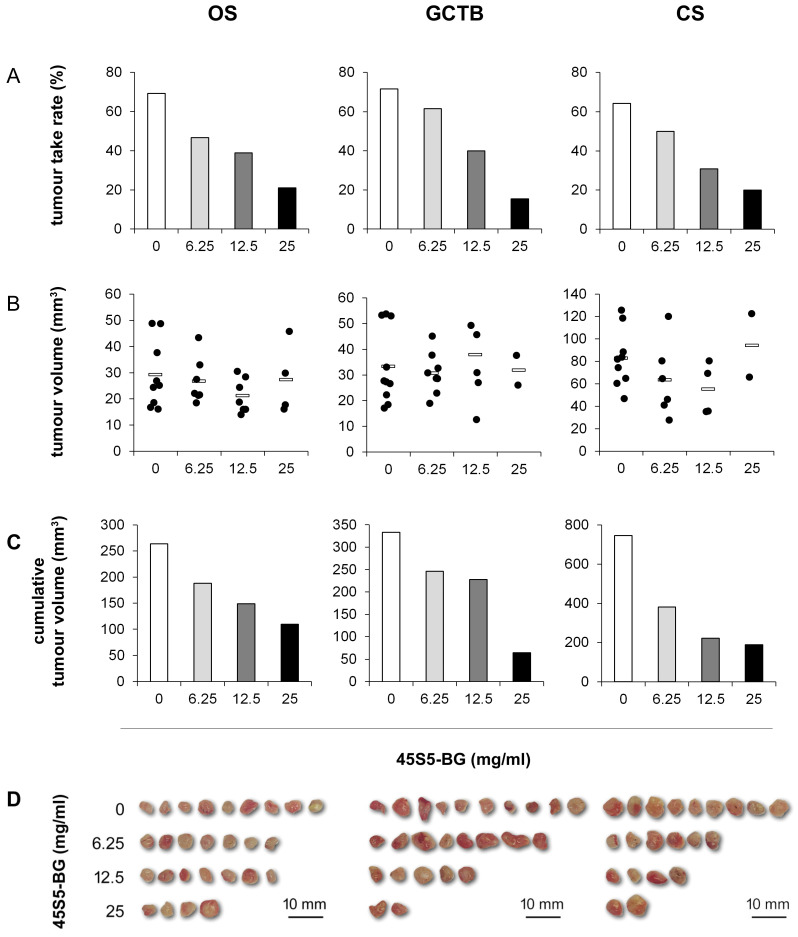
45S5-BG inhibits tumour formation in vivo. OS, GCTB and CS cells were co-transplanted with 45S5-BG at the indicated concentrations onto the CAM of fertilised chicken eggs (*n* = 20 for each experimental group). Tumours that formed after seven days were resected, and (**A**) the tumour take rate, (**B**) the volume of the individual tumours (white rectangles indicate the mean tumour volumes), and (**C**) the cumulative tumour volume of the experimental groups were calculated. (**D**) Photographs of the resected tumours. Representative data from the OS cell line MNNG-HOS, the CS cell line SW1353, and a primary GCTB cell line are shown.

Notably, the concentration of the BG particles had to be adapted to the experimental conditions. In contrast to the in vitro assays, where 10,000 cells are treated with BGs in a total volume of 200 µL, the CAM assay requires 10^6^ cells in a volume of 40 µL. Therefore, an identical ratio of BG particles to cells would require a 500-fold higher BG concentration in vivo than in the in vitro experiments. As it is not feasible to achieve such high BG concentrations, we used concentrations ranging from 6.25 to 25 mg/mL. At these concentrations, the density of the BG particles in the 3D constructs was comparable to that observed in the 2D in vitro setting. After seven days of incubation, the formed tumours were resected, and the tumour take rates and tumour volumes were calculated. Notably, 45S5-BG inhibited tumour formation in a dose-dependent manner, regardless of the tumour type. After the transplantation of OS, GCTB, and CS cells, solid tumours formed in 69%, 71%, and 64% of all cases. When the cells were co-transplanted with 45S5-BG (25 mg/mL), the tumour take rates decreased to 21%, 15%, and 20% (Figure 7A). In contrast to the number of tumours, the volume of the individual tumours did not change significantly after the addition of 45S5-BG (Figure 7B,D). Nevertheless, the cumulative tumour volumes, which represent the total volume of all tumours that formed within an experimental group, decreased significantly in a dose-dependent manner when 45S5-BG was added (Figure 7C).

## 3. Discussion

Treatment of bone tumours remains a challenging issue, and there is still a lack of fundamental progress in optimizing current therapies. It has been more than 40 years since the introduction of neoadjuvant chemotherapy improved the 5-year survival rate of OS patients from 20% to 65%. However, despite the recent advances in molecular biology, surgical techniques, and new treatment protocols, further improvements are limited and the survival rate especially for patients with metastatic disease remains very poor [30,31]. Similarly, the treatment of CS remains challenging due to its poor response to chemo- and radiation therapy, resulting in poor survival rates [32]. Although the survival rates are only a minor issue for the semi-malignant giant cell tumour of bone, it is the high incidence of recurrence and bone destruction that needs to be addressed to improve the outcomes for this tumour [21]. Therefore, there is an urgent need to improve the current treatment strategies for multiple types of very biologically different bone tumours.

We previously observed an unexpected BG-mediated cytotoxicity towards GCTB cells, suggesting a potential therapeutic use of BGs for the treatment of this and possibly other bone tumours. Thus, the objective of this study was to evaluate the suitability of BGs for such a universal therapeutic approach that can treat bone tumours in general. To be effective and ideally free of side effects, new therapeutic strategies should target specific properties of the tumour cells that are not present or will not be affected in healthy cells. To investigate whether and to what extent non-malignant cells would be affected by a BG-based therapy approach, we included 15 primary non-malignant cell lines in this study. These cell lines represent the most common cell populations within the sites of tumorigenesis.

With 45S5-BG, the first bioactive glass synthesised by L. Hench and extensively used in various biomedical applications, particularly in bone tissue engineering, we demonstrated a strong cytotoxic effect on bone tumour cells derived from OS, CS, and GCTB in vitro and in vivo. Using a CAM assay as an experimental model for the simulation of an in vivo environment, we could demonstrate that all xenografts maintained the characteristics of the original tumours and retained specific markers. BG treatment resulted in a significant and concentration-dependent reduction in tumour formation. Of note, this effect was independent of the tumour type, thereby confirming our in vitro data.

These data strongly support our hypothesis that 45S5-BG and probably other bioactive glasses represent promising candidates for the development of new and effective bone tumour therapies. Importantly, the viability of the investigated control cells was not or much less affected by a BG treatment, indicating that a BG-based therapy approach will be largely free of side effects. Although HOBs showed a moderate reduction in cell viability when treated with high BG concentrations, the beneficial effects of BGs on osteoblast differentiation and migration as well as the clinical applications of BGs for bone repair and tissue engineering have already been described in multiple studies [33,34]. These data indicate that BGs might be used for a combined therapy targeting tumour cells while simultaneously promoting bone healing, possibly after modifications that further reduce cytotoxicity towards HOBs. Given that BGs have also been demonstrated to promote angiogenesis, a process known to facilitate tumour formation, it is necessary to investigate whether this property may counteract the BG-mediated cytotoxicity. However, the cytotoxic effect of BGs occurs rapidly, while the formation of new vessels is a much slower process that is unlikely to prevent cell death. This assumption is supported by the results obtained in the in vivo environment of the CAM assay, which showed a significant reduction in tumour formation in response to BG treatment.

The observation that cytotoxicity increases as BG particle size decreases suggests that the cytotoxic effect may be influenced by the surface area of the particles, their absorption by the cells, or both. In fact, using electron and confocal microscopy, the internalisation of nano- and micrometre-sized BG particles by osteoblasts has already been demonstrated [16]. BG particles with smaller sizes (<0.2 µm) were found to be phagocytised by lysosomes, while larger particles (0.2–1 µm) were supposed to escape into the cytoplasm, leading to disruption of the F-actin cytoskeleton and lysosomal injury. The release of lysosomal enzymes is finally supposed to induce the observed cell death. The importance of the lysosomal injury for mediating the cytotoxic effect has also been shown in mouse macrophages after exposure to silica particles [35]. It has further been shown that physical parameters like size, shape, and surface charge affect the internalization of such particles [36,37]. To investigate whether, in addition to the particle size, the surface texture of the BGs plays a decisive role in the observed cytotoxicity, we analysed the influence of 45S5-BG particles on cell viability after various pre-treatments of the BG. One approach was to accelerate the demineralization process and the dissolution of the BG by using an acid etching method, that is also known to induce the release of ions such as Na^+^ and Ca^2+^. We further modulated the BG surface by heat treatment of the particles to achieve a surface cleaning. Finally, 45S5-BG was incubated in SBF, leading to the formation of a hydroxycarbonate apatite layer that is comparable to that found in vivo where it promotes bonding to hard tissue. Although significant in the case of GCTB cells, the described surface variations showed only minor effects on cytotoxicity. These observations were unexpected as we had shown in previous studies that a direct cell–BG contact is required to mediate the cytotoxic effect, suggesting that the surface texture is also important [19].

A significantly greater improvement in cytotoxicity, independent of tumour type, was achieved when the particles were subjected to an additional sintering process. Sintering significantly improved the cytotoxic effect of 45S5 particles > 25 µm, regardless of their surface pre-treatment. Sintering of the BGs has been shown to induce a partial crystallisation of the initially amorphous material that is accompanied by weight loss, shrinkage, and densification of the material. It has been proposed that the interfaces between the glass matrix and the crystallites formed by the sintering process facilitate a modulation of BG degradation. It has been further hypothesised that the ion exchange takes place at distinct micro-locations within the crystalline phases, and that this process results in the collapse of the crystalline particles into very fine grains [25,38]. This process may explain our observation that larger particles, initially less effective, gain significant cytotoxicity through sintering. This finding also agrees with our earlier study mentioned above, which also showed that cytotoxicity cannot be mediated by BG conditioned media containing the dissolution products, by BG-induced changes of the pH of the cell culture medium, or by BGs separated from the cells in a trans-well system [19]. Our studies on bio-inert particles derived from microscope slide glass, that demonstrate a cytotoxic effect comparable to that of 45S5-BG, finally confirmed our assumption that it is essentially the particle size, and less the chemical composition or surface properties, that mediates cytotoxicity towards tumour cells.

Although we have previously identified a mitogen-activated protein kinase (MAPK)-dependent but caspase-independent induction of autophagy in response to BG treatment in GCTB [18,19], the precise mechanisms that are responsible for the observed cell type-specific cytotoxicity still remain unknown and are the subject of our current research. One potential explanation is that damage-associated molecular patterns (DAMPs) may be involved in a cell-type-specific manner. DAMPs, also referred to as alarmins, are released from damaged or dying cells and function as endogenous danger signals. The group of known members includes the high-mobility group box protein 1 (HMGB1), cytochrome C, and the calcium-binding protein S100A. It is established that DAMPs can induce the production of pro-inflammatory cytokines, including IL-6. In the previous studies, we observed a rapid loss of intracellular HMGB1 followed by an increase in IL-6 expression in GCTB cells after BG treatment [18,19]. It can thus be postulated that DAMPs, including HMGB1, as well as IL-6, may be involved in BG-mediated cell death. Furthermore, DAMPs trigger their responses after binding to the receptor for advanced glycation end products (RAGE) or toll-like receptors (TLRs). TLR activation, in turn, induces the production of pro-inflammatory cytokines, chemokines, and interferon (IFN)-inducible genes. A whole genome gene expression analysis conducted in our previous study demonstrated the upregulation of multiple IFN-inducible genes in response to BG treatment, as well as a notable elevation in the expression of several transcription factors exclusively in neoplastic GCTB cells [18]. These findings suggest that the DAMP–TLR axis may play a role in BG-mediated cytotoxicity. The differential expression of key players involved in these processes may contribute to the observed cell type specificity.

In addition, the known acidic microenvironment of tumours may contribute to the cell type specificity. The dissolution of BGs and the formation of an hydroxyapatite layer have been demonstrated to increase significantly at lower pH values [39]. Consequently, the altered ion release profile and surface structure might then influence the cytotoxic effect. However, whether and which of these factors actually mediate BG-induced cell death in bone tumour cells needs to be investigated in further studies.

## 4. Materials and Methods

### 4.1. Origin of Cell Lines

Cell lines from three different tumour types and three different control tissues were used in this study. To analyse the effects of BGs on bone tumour cells, we used the giant cell tumour of bone (GCTB), osteosarcoma (OS), and chondrosarcoma (CS) cell lines (*n* = 5 each). As control cells primary bone marrow-derived mesenchymal stromal cells (BMSC), human osteoblast-like cells (HOB), and human chondrocytes (CHO) were analysed (*n* = 5 each).

The following five commercially available human osteosarcoma cell lines and two chondrosarcoma cell lines were used in this study: HOS 143B (Sigma-Aldrich, Taufkirchen, Germany), CAL-72 (DSMZ, Braunschweig, Germany), MNNG-HOS, Saos-2, and U2OS (Cell Line Service GmbH, Eppelheim, Germany), SW1353 (Cell Line Service GmbH), and CAL-78 (DSMZ). Authentication of these cell lines was performed by STR analysis by the companies. In addition, five primary GCTB and three primary CS cell lines were isolated from tumour biopsies obtained from the patients who underwent surgery at the Heidelberg Orthopedic University Hospital. Primary HOB and CHO cell lines were isolated from patients with diagnosed osteoarthritis of the knee who underwent a complete knee replacement surgery. Primary BMSCs were isolated from the bone marrow of patients that underwent primary hip arthroplasty at the Heidelberg Orthopedic University Hospital.

### 4.2. Isolation of Primary Cell Lines

For the isolation of GCTB cell lines, the tumour tissue was cut into small fragments, washed with PBS and then digested with 1.5 mg/mL collagenase B (Thermo Fisher Scientific, Karlsruhe, Germany) diluted in Dulbecco’s Modified Eagle Medium (DMEM) high glucose supplemented with 10% foetal calf serum (FCS) (Bio & Sell GmbH, Nürnberg, Germany) and 100 U/mL penicillin/streptomycin (Sigma-Aldrich) for 3 h at 37 °C. The cell suspension was washed twice with PBS and seeded in DMEM culture medium. After 48 h, the cells were treated with Trypsin/EDTA (Sigma-Aldrich), and the detached stromal cells were transferred to a new culture flask, separating them from the giant cells that remained attached to the plastic. After three further passages, the neoplastic stromal cell population was free of remaining giant cells and histiocytes and checked for the existence of the characteristic H3F3A-G34W mutation by restriction site mutation analysis as previously described [40].

Primary CS cell lines were isolated in a comparable way, except that the cartilaginous tumour tissue was digested overnight with collagenase B and the cells were cultured in RPMI 1640 cell culture medium containing 10 mM HEPES (Thermo Fisher Scientific) supplemented with 100 U/mL penicillin/streptomycin (Sigma-Aldrich) and 10% FCS (Bio & Sell GmbH).

For the isolation of primary HOB and CHO cell lines, the cartilage and bone areas of the material obtained after a complete knee replacement surgery were separated using a scalpel. Cartilage and bone chips were cut into small pieces (<1 mm for cartilage and 1–4 mm for bone) and digested with 1.5 mg/mL collagenase B (Thermo Fisher Scientific) overnight at 37 °C with gentle rotation. After washing with PBS, cells were pelleted by centrifugation, resuspended in culture medium and seeded in gelatin-coated culture flasks. At 80% confluency, cells were passaged, and the bone fragments were discarded. HOB culture medium consisted of DMEM low glucose (Thermo Fisher Scientific) containing 1 g/L glucose, 10% FCS (Bio & Sell GmbH), 100 U/mL penicillin/streptomycin (Sigma-Aldrich), and 0.17 mM ascorbic acid 2-phosphate (Sigma-Aldrich). Primary CHO cell lines were cultured in RPMI 1640 cell culture medium containing 10 mM HEPES (Thermo Fisher Scientific) supplemented with 100 U/mL penicillin/streptomycin (Sigma-Aldrich) and 10% FCS (Bio & Sell GmbH).

Primary BMSCs were isolated from bone marrow as described previously [18] and cultured in gelatin-coated cell culture flasks (0.1%). The BMSC culture medium consisted of DMEM (Thermo Fisher Scientific) high glucose supplemented with 10% fetal calf serum (FCS) (Bio & Sell GmbH), 1% non-essential amino acids (NEAA), 50 µM β-mercaptoethanol, 100 U/mL penicillin/streptomycin, and 4 ng/mL fibroblast growth factor 2 (all Sigma-Aldrich).

### 4.3. BG Production

With a chemical composition SiO_2_ (46.1%), CaO (26.9%), Na_2_O (24.4%), and P_2_O_5_ (2.6%) in mol%, 45S5-BG was produced by the melt-quenching method as detailed elsewhere [19]. The glass was ground with a planetary ball mill (Retsch, Haan, Germany) and different particle size ranges were obtained using sieves of 25, 45, 90, 125, and 250 µm. Moreover, a group of sintered BG powders was considered, in which particles of less than 25 µm were compacted into pellets with a hydraulic press and sintered at 1050 °C for 2 h at a heating rate of 2 °C/min. Subsequently, grinding and sieving followed, as previously described. Furthermore, the following three independent surface treatments were carried out on sintered and non-sintered BG particles to obtain samples with different topographic features: (i) A thermal treatment was performed for 30 min at 400 °C; a relatively low temperature was selected to perform a surface cleaning and to avoid structural changes in the glasses. (ii) An acid etching process with 4M hydrochloric acid (HCl, AnalaR NORMAPUR^®^) (VWR^®^, Leuven, Belgium) was carried out on the BG particles for 30 min in an ultrasonic bath. (iii) BG particles were incubated in simulated body fluid (SBF) for 7 days at 37 °C using a vertical rotator. The SBF solution was prepared according to the protocol of Kokubo et al. [41], and it was refreshed two times during the incubation period. Additionally, commercial microscope glass slides (clear, float glass, Cat. No. 631-1550, VWR^®^, Leuven, Belgium) were ground and sieved following the same protocol as the BG particles. This glass was considered as reference of inert material.

The characterization of the glass particles was carried out by means of scanning electron microscopy at a voltage of 1.5 kV (SEM, Auriga, Carl-Zeiss, Jena, Germany), particle size measurements by laser diffraction (Mastersizer 3000, Malvern Panalytical, Malvern, UK), and X-ray diffraction (XRD, Miniflex 600 HR, Rigaku, Neu-Isenburg, Germany), with a step size of 0.02° over a 2θ range from 10° to 60°.

### 4.4. Cytotoxicity Assay

A WST-1 assay (water-soluble tetrazolium salt) (Santa Cruz Biotechnology, Heidelberg, Germany) was used to quantify the cytotoxicity of BGs. The BG to be tested was resuspended in cell culture medium at 2-fold concentration before pipetting 100 µL into the wells of 96-well plates. Immediately thereafter, 100 µL of cell culture medium containing 10,000 cells was added to each well. At the end of the incubation period, the medium was replaced with 100 µL of WST-1 reagent diluted 1:10 in cell culture medium and incubated at 37 °C for 120 min. Finally, the optical absorbance of the supernatants was determined in a plate reader (Autobio-Phomo, Anthos Microsystems, Friesoythe, Germany) at 450 nM with a reference wavelength of 600 nM. Wells without cells were used as blanks and subtracted from the experimental sample wells. All measurements were performed in duplicate.

### 4.5. Lens Culinaris Agglutinin Staining

For the detection of chicken cells and blood vessels, 5 µm sections of paraffin-embedded tumour xenografts were used. After deparaffinisation in Roti-Histol (Carl-Roth, Karlsruhe, Germany) and rehydration in isopropanol (100%, 96%, 70%, and 50%, 5 min each) the tissue sections were blocked in 5% bovine serum albumin (BSA) (Sigma-Aldrich) diluted in PBS for 15 min at room temperature. After a washing step in PBS, the sections were incubated with biotinylated Lens Culinaris Agglutinin (5 µg/mL in PBS) (Linaris, Dossenheim, Germany) for 30 min and washed twice in PBST (PBS supplemented with 0.5% Tween 20) and once in PBS. Detection of bound and biotinylated Lens Culinaris Agglutinin was performed by incubation in AB reagent (Avidin D/biotinylated alkaline phosphatase) (Linaris, Dossenheim, Germany) for 30 min. Sections were washed again as described above and incubated in alkaline phosphatase substrate (ImmPACT Vector Red) (Linaris) for 20 min. After a final wash in distilled H_2_O, sections were counterstained with Methyl Green (Linaris), mounted using Neomount (Sigma-Aldrich), and imaged using a Keyence BZ-X800 microscope (Keyence, Neu-Isenburg, Germany).

### 4.6. ALU In Situ Hybridisation

A Digoxigenin-labelled probe, specific for a human ALU repetitive DNA sequence, was generated by polymerase chain reaction (PCR) as previously described using the following primer: ALU-F 5′-CGAGGCGGGTGGATCATGAGGT-3′, ALU-R 5′-TTTTTTGAGACGGAGTCTCGC-3′ [29]. Paraffin-embedded tumour sections were deparaffinised in Roti-Histol (Carl Roth, Karlsruhe, Germany), rehydrated in isopropanol, and digested with proteinase K (Jena Bioscience, Jena, Germany) diluted in PBS (5 µg/mL) for 15 min at 37 °C. After washing in PBS, the sections were prehybridised at 42 °C for 1 h in prehybridisation buffer containing 4 × saline sodium citrate (SSC), 50% deionised formamide, 1 × Denhardt’s solution, 5% dextrane sulphate (all Sigma-Aldrich) and 100 µg/mL salmon sperm DNA (Thermo Fisher Scientific). The prehybridisation buffer was replaced by fresh buffer containing 0.2 ng/µL of PCR-generated digoxigenin-labelled ALU probe before heating the sections at 95 °C for 5 min to denature the probe and the target DNA. The samples were hybridised for 16 h at 42 °C in a wet chamber, washed twice for 5 min in 2 × SSC buffer at room temperature and twice for 10 min at 42 °C in 0.1 × SSC. Finally, anti-Digoxigenin alkaline phosphatase-conjugated Fab fragments (Roche Diagnostics, Mannheim, Germany) and the substrate NBT/BCIP (Linaris) were used to detect the DNA-bound probe. Sections were counterstained with Methyl Green (Linaris) and imaged using a Keyence BZ-X800 microscope (Keyence).

### 4.7. Immunohistochemistry

Prior to the detection of the various antigens, an antigen retrieval step was performed using Dako Target Retrieval Buffer pH 6 (Dako, Hamburg, Germany). The deparaffinised and rehydrated tissue sections were heated to 121 °C for 5 min in retrieval buffer in a pressure cooker, cooled to room temperature, and blocked with PBS supplemented with 5% BSA (Sigma-Aldrich). Sections were incubated overnight at 4 °C with the following primary antibodies diluted in 1% BSA (Sigma-Aldrich): Anti-human H3.3-G34W (RevMab Biosciences, San Francisco, CA, USA) diluted 1:200, anti-human Collagen type II (MP Biomedicals, Eschwege, Germany) diluted 1:1000 and anti-human Prim1 (Proteintech, Planegg-Martinsried, Germany) diluted 1:100. After three washes in Tris-buffered saline (TBS), the bound antibodies were detected using the BrightVision plus kit (VWR International, Darmstadt, Germany) according to the manufacturer’s instructions. ImmPACT Vector Red (Linaris) was used as the substrate. Samples were counterstained with Methyl Green, mounted with NeoMount (VWR International, Darmstadt, Germany), and imaged using a Keyence BZ-X800 microscope (Keyence).

### 4.8. Chicken Chorioallantoic Membrane (CAM) Assay

Fertilised Leghorn chicken eggs (Geflügelzucht Hockenberger, Eppingen, Germany) were placed into a motor breeder in an upright position with the pointed side facing downwards. The eggs were incubated at 37.8 °C and 70% humidity with permanent agitation. After three days, 3 mL of albumen was removed with a 20-gauge needle attached to a 5 mL syringe to prevent the CAM from attaching to the eggshell. A Leukosilk tape (BSN medical GmbH, Hamburg, Germany) was applied to the top of the egg before a window of approximately 1.5 cm diameter was cut into the eggshell. The window was resealed with Leukosilk tape, and the eggs were further incubated in a horizontal position without agitation until embryonic developmental day 9 (EDD 9).

For the transplantation of the tumour cells, the window was opened again and a sterile silicone ring with an inner diameter of 9 mm was placed onto the CAM. The CAM area within this ring was gently lacerated using a 30-gauge needle, before 40 µL of a cell/matrix suspension was inoculated. This suspension consisted of 1 × 10^6^ cells in 20 µL DMEM cell culture medium with or without the addition of 45S5-BG mixed with an equal volume of Cultrex BME Type 3 matrix, a soluble form of basement membranes (AMS Biotechnology, Frankfurt, Germany). The eggs were further incubated for seven days until the tumour xenografts were resected after humane euthanasia of the embryo by injection of 50 µL of the pentobarbital Narcoren into the chicken vasculature. Each experimental group consisted of at least 20 eggs. Embryos that have died before EDD 16 and contaminated eggs were excluded from the study. The tumours were photographed, and the longitudinal and transverse diameters were quantified using ImageJ software 1.48v (https://imagej.net/ij/ accessed on 12 April 2024). The diameters were then used to calculate the tumour volumes using the following formula: volume = 4/3 × π × r^3^ (r = ½ × √ of diameter 1 × diameter 2) [42]. The tumour take rates correspond to the percentage of tumours formed within an experimental group of eggs with vital embryos.

### 4.9. Fluorescent Live/Dead Staining

After treatment with 45S5-BG, the cells were stained with a mixture of 1 µM Calcein-AM (Biozol, Eching, Germany, 1 µg/mL Hoechst 33342 (Biomol, Hamburg, Germany) and 1 µg/mL propidium iodide (Thermo Fisher Scientific) in culture medium for 20 min at 37 °C. After two washes in HBS buffer, images were taken using a Keyence BZ-X800 microscope (Keyence).

### 4.10. Statistics

Descriptive statistics, such as mean and standard deviation, were calculated using SPSS software (Version 25; IBM, Armonk, NY, USA). The nonparametric Mann–Whitney U test was used to compare experimental groups, with *p*-values < 0.05 regarded as statistically significant. Reported *p*-values are two-sided. Data are presented as mean ± standard deviation. The given number of n always refers to the number of biological replicates (cell lines or eggs in CAM assays).

## 5. Conclusions

To our knowledge, we demonstrated, for the first time, a strong cytotoxic effect of 45S5-BG particles on the tumour cells derived from three different bone tumour entities in vitro and in vivo. This effect was concentration- and time-dependent and was absent or very weak in non-malignant cells. A further novelty of our study is the analysis of the diversity of bone tumours and inter-patient variabilities through the examination of 30 individual cell lines, which is crucial for the evaluation of a potential therapeutic application of BGs. A significant modulation of the observed cytotoxic effect could be achieved by altering the particle size or performing a sintering step during the production of the BGs. Given the known beneficial effects of BGs on bone regeneration, our findings suggest that 45S5-BG and other BGs hold great potential for the development of new and effective therapies to target bone tumours while minimising unwanted side effects.

Although our data indicate that cytotoxicity is independent of bioactivity, the use of BGs could allow for a combined therapy approach that targets bone tumour cells and simultaneously contributes to bone healing. Our current research focuses on the identification of the molecular mechanisms that finally lead to cell death and particularly those that confer tumour cell specificity. This knowledge may enable the design of even more potent BG particles.

## Figures and Tables

**Table 1:** Particle size distribution in µm.

Size Range	Particle Size Distribution	45S5-BG Non-Sintered	45S5-BG Sintered	Microscope Slide Glass
<25 µm	D10	8.5 ± 0.5	5.6 ± 0.2	2.5 ± 0.1
	D50	17.5 ± 0.8	17.0 ± 0.2	6.8 ± 0.6
	D90	32.8 ± 2.0	36.2 ± 2.4	20.2 ± 2.7
25–45 µm	D10	27.9 ± 0.5	25.4 ± 0.3	19.7 ± 0.1
	D50	46.9 ± 0.4	43.5 ± 0.1	25.8 ± 0.2
	D90	75.6 ± 0.3	70.6 ± 0.1	44.4 ± 0.1
45–90 µm	D10	57.2 ± 0.2	58.0 ± 0.2	36.6 ± 0.1
	D50	90.7 ± 0.3	90.9 ± 0.2	63.8 ± 0.4
	D90	142 ± 0.3	139.5 ± 0.2	125 ± 0.6

## Data Availability

Data will be made available upon reasonable request from the corresponding author.
